# Anifrolumab—A Potential New Systemic Sclerosis Treatment

**DOI:** 10.3390/jcm15031104

**Published:** 2026-01-30

**Authors:** Mislav Radić, Petra Šimac Prižmić, Tina Bečić, Hana Đogaš, Dijana Perković, Josipa Radić, Damir Fabijanić

**Affiliations:** 1 Department of Internal Medicine, Division of Rheumatology, Allergology and Clinical Immunology, Center of Excellence for Systemic Sclerosis in Croatia, University Hospital of Split, 21000 Split, Croatia; psimac@kbsplit.hr (P.Š.P.); dperkov@kbsplit.hr (D.P.); 2 Internal Medicine Department, School of Medicine, University of Split, 21000 Split, Croatia; jradic@kbsplit.hr (J.R.); dfabijan@mefst.hr (D.F.); 3 Cardiovascular Disease Department, University Hospital of Split, 21000 Split, Croatia; tbecic@kbsplit.hr; 4 Department of Neurology, University Hospital of Split, 21000 Split, Croatia; hdogas@kbsplit.hr; 5 Department of Internal Medicine, Division of Nephrology, Dialysis and Arterial Hypertension, University Hospital of Split, 21000 Split, Croatia

**Keywords:** systemic sclerosis, anifrolumab, type I interferon, interferon signature, precision medicine

## Abstract

**Background/Objectives:** Systemic sclerosis (SSc) is a rare autoimmune disease characterized by chronic inflammation, microvascular injury, and fibrosis of the skin and internal organs. Although there are therapies, there is a need for treatments targeting early pathogenic mechanisms. Type I interferons (IFN-I) are key mediators linking immune dysregulation to vascular and fibrotic damage in SSc. This review summarizes the current evidence supporting IFN-I blockade with anifrolumab as a novel therapeutic strategy. **Methods:** A narrative review of preclinical, translational, and emerging clinical studies was conducted to evaluate the role of IFN-I signaling in SSc and the therapeutic potential of anifrolumab. Particular focus was placed on the IFN signature, upregulation of interferon-stimulated genes (ISGs), and the association with disease activity and organ involvement. **Results:** Anifrolumab, a fully human monoclonal antibody targeting the IFN-I receptor subunit 1 (IFNAR1), inhibits the signaling of all IFN-I isoforms, suppressing downstream JAK–STAT activation and ISG expression. Mechanistic data suggest that IFNAR blockade modulates vascular injury, immune activation, and fibrosis. Early findings and ongoing trials indicate potential benefits, particularly in patients with a high IFN signature or rapidly progressive cutaneous and cardiac disease. **Conclusions:** The current evidence supports IFN-I pathway inhibition as a promising approach in SSc. Ongoing trials will help to determine the clinical efficacy, safety, and optimal patient selection for anifrolumab in this rare but severe disease.

## 1. Introduction

Systemic sclerosis (SSc) is a chronic autoimmune disease with a wide spectrum of clinical manifestations, characterized by a triad of immune dysregulation, vascular pathology, and fibrosis. Due to the heterogeneity of organ involvement and variability in the disease course, the treatment of SSc remains a major clinical challenge [1]. Current therapies, including immunosuppressants and antifibrotic agents, do not adequately address all domains of SSc and have limited impact on the long-term disease progression. This underscores the unmet need for therapeutic strategies that more directly target the upstream immune pathways driving fibrosis and vascular dysfunction [2,3,4].

Advances in the understanding of SSc pathogenesis have highlighted type I interferons (IFN-I) as critical mediators in both tissue fibrosis and vascular dysfunction. Several studies have identified a prominent “interferon (IFN) signature”, marked by the elevated expression of interferon-stimulated genes (ISGs) in the blood and tissues of SSc patients, particularly those with diffuse skin involvement and internal organ complications [5,6,7]. These findings strongly support the rationale for therapeutically targeting the IFN-I pathway.

Anifrolumab is a fully human monoclonal antibody that binds to the type I interferon receptor subunit 1 (IFNAR1), thereby blocking all IFN-I isoforms and inhibiting downstream Janus kinase-signal transducer and activator of transcription (JAK-STAT) signaling. It has demonstrated clinical efficacy and a favorable safety profile in systemic lupus erythematosus (SLE), especially in patients with a high IFN signature [8,9]. Given the mechanistic overlap between SLE and SSc, anifrolumab is now being explored as a potential immunomodulatory therapy in SSc [10]. Historically, most drugs used in SSc, including immunomodulatory and antifibrotic therapies, have been ‘borrowed’ from other indications, based on shared pathogenic mechanisms, rather than developed specifically for SSc [11]. Anifrolumab represents a contemporary example of this approach, having been developed for SLE, with its potential application in SSc emerging from the overlapping interferon-driven disease biology [6]. In this review, we summarize the current data and explore the future potential of anifrolumab in the treatment of SSc.

## 2. Materials and Methods

This narrative review was based on a comprehensive literature search performed in PubMed (https://pubmed.ncbi.nlm.nih.gov/, accessed 20 October 2025), Scopus (https://www.scopus.com/, accessed 20 October 2025), and ClinicalTrials.gov (https://clinicaltrials.gov/, accessed 20 October 2025), covering publications available up to October 2025, using the terms systemic sclerosis, IFN-I, IFNAR blockade, and anifrolumab. The search focused on preclinical, translational, and clinical studies addressing type I interferon signaling in SSc and the therapeutic potential of IFNAR blockade, including original research articles, clinical trials, and relevant review papers published in English. The reference lists of selected publications were manually screened to identify additional relevant sources. A formal systematic analysis was not performed, given the limited availability of clinical data and the absence of completed randomized controlled trials specifically evaluating anifrolumab in SSc. Therefore, the findings are narratively summarized to provide a comprehensive overview of the current mechanistic and translational evidence. No new patient data, animal experiments, or datasets were generated; therefore, ethical approval and informed consent were not required. All the analyzed information was derived from previously published peer-reviewed articles.

## 3. Type I Interferons in Systemic Sclerosis Pathogenesis

Although the role of IFN-I in the pathogenesis of SSc is well recognized, the precise mechanisms underlying the IFN signature and its contribution to key pathophysiological processes in SSc remain incompletely understood [6]. IFN-I includes cytokines that are released into the extracellular space as part of the innate immune response to various viral pathogens. In addition to mediating the antiviral response, they also orchestrate both innate and adaptive immune mechanisms [12,13]. Earlier studies have shown that a significant proportion of patients with SSc exhibit increased IFN-I activity. According to several reports, a detectable ISG signature is observed in up to approximately 50% of patients, particularly in those in the early phase of the disease [14,15,16,17,18,19]. Associations between the IFN signature and IFN-inducible chemokines with autoantibody profiles have also been reported, whereas the findings regarding differences between limited cutaneous and diffuse cutaneous SSc remain conflicting [20,21]. Dysregulation of ISGs has been identified in the skin of patients with SSc and has also been reported in lung tissue; however, the pulmonary findings remain heterogeneous. Despite this variability, the altered expression of interferon-related genes has been documented in lungs affected by interstitial lung disease (ILD) [22,23,24].

Chronic stimulation of the innate immune system by endogenous and exogenous nucleic acids has been implicated in the activation of IFN-I pathways in SSc [6]. Toll-like receptors (TLRs), acting as pattern-recognition receptors, initiate intracellular signaling cascades that lead to the activation of interferon regulatory factors and the subsequent induction of IFN-α and IFN-β [25]. Among immune cells, plasmacytoid dendritic cells (pDCs) represent the principal source of IFN-α [26]. Increased infiltration of pDCs has been demonstrated in the skin of patients with SSc and is associated with enhanced local IFN-α activity. CXCL4 (platelet factor 4), which is markedly elevated in SSc, forms complexes with microbial or self-DNA that activates TLR9 within pDCs, thereby further stimulating IFN-α production. Elevated levels of CXCL4 correlate with pulmonary fibrosis and pulmonary hypertension. Autoantibodies to CXCL4, which are present in very early SSc, further stimulate pDCs, creating a self-perpetuating cycle of IFN activation [27,28]. Infectious agents such as Epstein–Barr virus (EBV) also activate pathways that include TLRs. Specifically, infection of monocytes by EBV results in enhanced TLR9 activity; these infected monocytes can subsequently transmit EBV to endothelial cells in SSc skin, causing vascular and endothelial injury [29]. The role of mitochondrial DNA (mtDNA) in the IFN signature is also important. Under conditions of mitochondrial stress or damage, fragmented mtDNA can be released into the extracellular space, where it acts as a damage-associated molecular pattern capable of engaging innate immune pathways linked to type I interferon signaling. In line with this concept, increased levels of circulating mtDNA have been reported in patients with SSc and have been shown to be associated with the presence and progression of ILD, supporting a potential link between mitochondrial dysfunction, IFN activation, and organ involvement [30,31]. These observations indicate that multiple endogenous and exogenous stimuli cross on the same signaling cascade, sustaining chronic IFN production. Once IFN is released, it binds to the IFN-α receptor (IFNAR1/2), resulting in activation of the JAK-STAT and tyrosine kinase 2 signaling pathways, which are aberrant in SSc patients [32]. Together with IRF9, they form a crucial transcriptional complex known as ISGF3, which drives the persistent secretion of interferon-inducible chemokines (CXCL9, CXCL10, CXCL11) and effector proteins such as myxovirus-resistance protein A (MxA). Collectively, these mediators sustain inflammation and vascular remodeling [33].

The association between IFN and fibrosis has been well demonstrated in vivo, where IFN-activated monocytes and macrophages secrete profibrotic mediators that stimulate fibroblast proliferation and collagen synthesis [27]. Furthermore, chronic activation of pDCs promotes fibroblast-to-myofibroblast transition through IFN-α and CXCL4 signaling [23]. Type I interferon-driven inflammatory pathways are associated with the reduced expression of endothelial transcription factors such as Fli-1, which are critical for maintaining vascular integrity and whose deficiency contributes to vasculopathy [34].

Interactions between IFN-I and profibrotic growth factors further promote fibrogenesis. IFN-I enhances transforming growth factor beta (TGF-β) activation and sustains extracellular matrix deposition, while TGF-β reciprocally amplifies TLR- and IFN-dependent transcriptional pathways in fibroblasts. Moreover, in patients with rapidly progressive ILD, increased activation of the IFN–TGF-β signaling axis has been demonstrated. Oxidative stress and hypoxia in SSc tissues also facilitate the endothelial-to-mesenchymal transition, providing another mechanism through which IFN-I-driven endothelial injury leads to the accumulation of activated myofibroblasts. These effects can be reduced by the inhibition of NADPH oxidase or mitochondrial reactive oxygen species production. Suppression of the IFN signature following high-dose immunosuppression and autologous stem-cell transplantation correlates with a clinical improvement in SSc patients [35,36,37].

Together, these findings indicate that the sustained activation of pDCs and other antigen-presenting cells by innate immune stimuli contributes to both increased IFN-I production and elevated ISG expression within vascular and mesenchymal tissues. Persistent IFN signaling promotes endothelial damage, perivascular inflammation, and fibroblast activation, establishing a self-amplifying loop of vasculopathy and fibrosis that defines SSc.

## 4. Mechanism of Action of Anifrolumab

By blocking IFNAR1, anifrolumab prevents the downstream signaling of all type I IFN isoforms, including IFN-α, IFN-β, IFN-ω, IFN-κ, and IFN-ε [38,39]. However, following IFNAR engagement, downstream JAK–STAT signaling is activated, leading to the transcription of ISGs [40]. As outlined above, the sustained activation of this pathway contributes to inflammation, vascular injury, and fibrosis in SSc. Excessive or chronic activation of this pathway has been implicated in tissue inflammation and autoimmunity, as demonstrated in SLE [39].

By binding specifically to the subdomain 3 of IFNAR1, anifrolumab prevents IFN-I interaction with the receptor. This blockade interrupts both autocrine and paracrine IFN signaling, halting the positive feedback loops that otherwise amplify interferon responses. In addition to neutralizing the receptor signaling, anifrolumab induces the rapid depletion of IFNAR1 from the cell surface, thereby reducing the receptor availability for subsequent stimulation [38,41]. In pDCs, this mechanism downmodulates STAT1 phosphorylation, suppresses ISG transcription, and decreases the secretion of pro-inflammatory mediators and costimulatory molecules, ultimately suppressing T-cell activation and B-cell differentiation into autoantibody-producing plasma cells [42]. Following intravenous administration, anifrolumab exhibits a dose-dependent clearance that decreases as the receptor occupancy becomes saturated. The approved 300 mg every 4 weeks regimen yields mean trough concentrations of approximately 15 µg/mL at a steady state and an effective terminal half-life of ≈18 days. The exposure increases more than proportionally between 100 and 1000 mg, indicating the saturation of IFNAR-mediated elimination at therapeutic levels. Body weight and baseline IFN-gene-signature status modestly influence the clearance, but neither factor warrants a dose adjustment [43]. In contrast, patients with active lupus nephritis demonstrate enhanced clearance and lower systemic exposure, suggesting that intensified dosing may be required in renal disease studies [44]. The pharmacodynamic effect of the IFNAR1 blockade is commonly assessed by suppression of the peripheral interferon-gene signature, a biomarker of IFN-I activity. Across phase II and III trials, treatment with anifrolumab 300 mg produced rapid (by week 4), profound (≈85–90%), and sustained neutralization of the 21-gene IFN signature for 52 weeks [38,45,46]. Neutralization correlated positively with clinical response indices such as the British Isles Lupus Assessment Group-based Composite Lupus Assessment and the Systemic Lupus Erythematosus Responder Index. Patients achieving the highest quartile of IFN-signature reduction displayed the highest likelihood of attaining key disease-activity endpoints. These data confirm that the near-complete inhibition of IFNAR signaling is necessary to disrupt the inflammatory transcriptional milieu underlying IFN-driven autoimmunity. However, as with other biologic agents that induce the profound inhibition of immune signaling pathways, this degree of IFNAR blockade may also be associated with an increased risk of adverse effects. Clinical experience from SLE trials indicates a higher incidence of viral infections, particularly herpes zoster, highlighting the need for careful safety monitoring when translating this approach to SSc [47,48,49].

The abovementioned findings have been consolidated through the phase III TULIP trials in SLE, which demonstrated that receptor blockade, rather than selective cytokine neutralization, yields the most comprehensive suppression of IFN-mediated pathology [48]. In the TULIP-2 study, anifrolumab 300 mg achieved a 16–17% absolute increase in disease activity response versus a placebo, along with significant reductions in glucocorticoid use and cutaneous disease severity [49]. Post hoc analyses confirmed consistent efficacy across demographic and serologic subgroups, with the highest benefit observed in patients exhibiting high baseline IFN signatures [45]. Biomarker studies further revealed the normalization of complement, reductions in anti-double-stranded DNA antibody titers, and broad attenuation of ISG-related chemokines such as CXCL10 and CXCL11 [47,48,49]. These findings highlight the relationship between the IFN-I blockade, transcriptional reprogramming, and clinical improvement.

**Figure 1:**
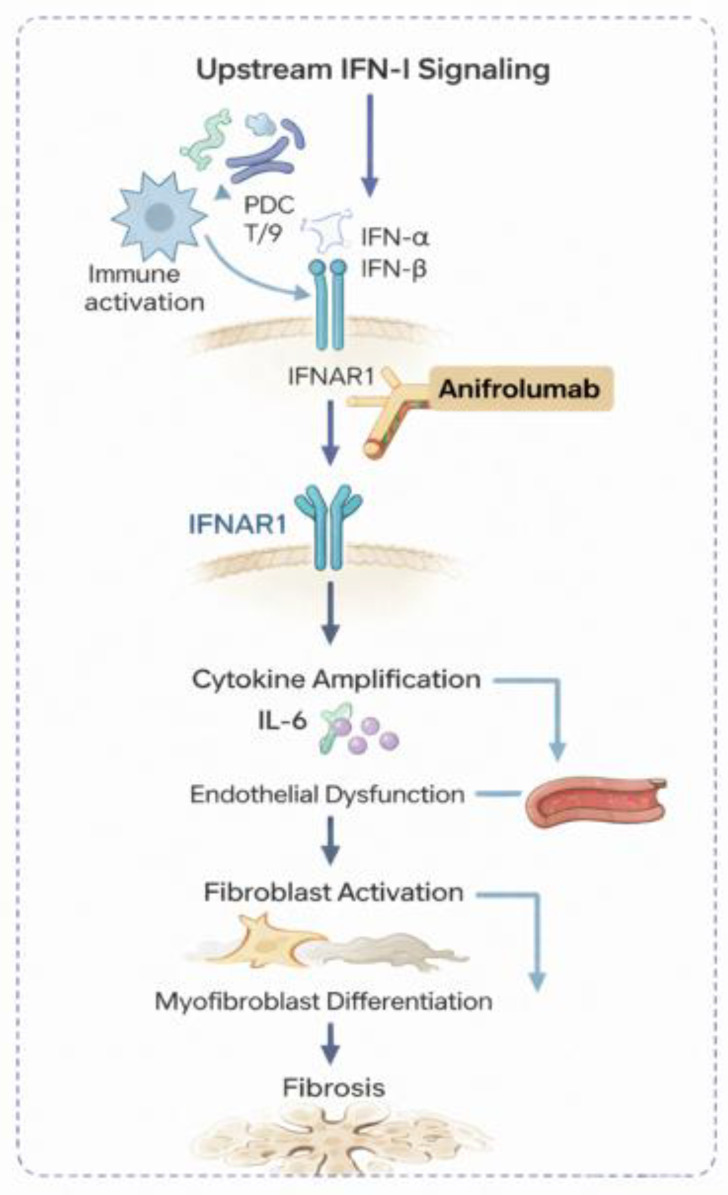
Schematic representation of the pathogenic cascade in systemic sclerosis and the proposed positioning of anifrolumab. Type I interferons act as upstream mediators linking innate immune activation to downstream cytokine amplification, vascular injury, and fibrotic remodeling. Anifrolumab blocks IFN-I signaling at the level of IFNAR1, thereby attenuating subsequent immune activation, endothelial dysfunction, and fibroblast activation. In contrast, other targeted agents act predominantly at downstream inflammatory or fibrotic stages of the disease process. Abbreviations: IFN-I, type I interferon; IFN-α, interferon-α; IFN-β, interferon-β; IFNAR1, interferon-α/β receptor subunit 1; IL-6, interleukin-6; pDC, plasmacytoid dendritic cell; TLR7/9, Toll-like receptor 7/9.

Collectively, anifrolumab acts by interrupting the core signaling axis of IFN-I activity. Through high-affinity binding to IFNAR1, the antibody productive receptor engagement induces receptor internalization and inhibits downstream JAK–STAT activation. The pharmacokinetic and pharmacodynamic profiles confirm sustained systemic receptor coverage with the 300 mg Q4W regimen, translating into the robust neutralization of IFN-stimulated gene expression and consequent clinical benefits [38,42,46]. In addition to the intravenous formulation, a subcutaneous version of anifrolumab is currently being evaluated in the ongoing DAISY trial (NCT05925803) in patients with diffuse cutaneous SSc [10,38]. This approach aims to improve the treatment convenience, maintain the comparable pharmacodynamic suppression of the interferon signature, and enable broader clinical application once efficacy and safety are confirmed [10]. The pathogenic triad of SSc and the upstream mechanism of IFN-I blockade by anifrolumab are illustrated in Figure 1.

## 5. Preclinical and Clinical Evidence in Systemic Sclerosis

Preclinical data on interferon-related signaling in SSc are limited but increasingly supportive of its translational relevance. The most compelling insights come from murine models of bleomycin-induced fibrosis, which recapitulate key features of human SSc by combining inflammation, vascular injury, and extracellular matrix accumulation. In these models, genetic ablation of key regulators of type I interferon signaling, such as interferon regulatory factor 7 (IRF7), resulted in the marked attenuation of dermal fibrosis and inflammatory cell infiltration. In particular, IRF7-deficient mice demonstrated reduced fibroblast activation, decreased α-smooth muscle actin expression, and suppression of profibrotic gene programs in response to bleomycin exposure. These findings support the concept that type I interferon-related signaling pathways act upstream of profibrotic cascades in SSc pathogenesis, providing a mechanistic rationale for therapeutic strategies targeting the interferon axis [50]. These murine models have limitations when extrapolating findings to human SSc due to the chronic, autoimmune, and multisystem nature of the disease itself, including immune dysregulation, vascular pathology, and long-term disease progression. Consequently, those preclinical data should be interpreted with caution when assessing the translational potential of the IFNAR blockade. These considerations highlight the need for complementary preclinical approaches and vigorous clinical trial data to better define the therapeutic relevance of anifrolumab in SSc.

Further preclinical evidence has emerged from pDC depletion studies, showing that the removal of IFN-α-producing pDCs protects against dermal fibrosis and attenuates disease development in murine models of scleroderma [51]. In parallel, CXCL4 has been identified as a key profibrotic mediator: pDC-derived CXCL4 can potentiate interferon signaling, while experimental CXCL4 deficiency or antibody-mediated blockade reduces bleomycin-induced fibrosis by limiting fibroblast-to-myofibroblast transition and collagen accumulation [51,52]. These animal studies collectively suggest that the pathological IFN–CXCL4 axis can be therapeutically targeted to disrupt both the inflammatory and fibrotic phases of SSc.

Given the strong pathophysiologic overlap between SSc and SLE, translational insights from SLE research have played a key role in accelerating the development of the interferon blockade. In SLE, multiple phase III trials (TULIP-1, TULIP-2) demonstrated that anifrolumab showed clinically meaningful reductions in disease activity, with TULIP-2 meeting its primary endpoint and post hoc analyses supporting improved sustained glucocorticoid tapering, with an acceptable safety profile [48,49]. Importantly, patients with a high baseline IFN signature exhibited the most pronounced clinical response [45]. Similar IFN-driven transcriptional profiles are present in SSc, especially in diffuse cutaneous subsets, which supports extrapolating the therapeutic benefit [22,53]. Furthermore, transcriptomic analyses of SSc skin biopsies have shown the normalization of interferon-regulated pathways following autologous stem-cell transplantation or effective immunosuppression, suggesting that IFN signature modulation correlates with disease improvement [36].

Drawing on this translational rationale, anifrolumab was first evaluated in SSc in a phase I dose-escalation study that demonstrated the dose-dependent suppression of circulating interferon-regulated transcripts and confirmed the pharmacodynamic target engagement [54]. Based on these results, the ongoing phase III DAISY trial (NCT05925803) is assessing the efficacy and safety of subcutaneous anifrolumab versus a placebo in adults with SSc. The study’s primary endpoint is the proportion of patients achieving a Revised Composite Response Index in Systemic Sclerosis (Revised-CRISS-25) response at week 52, while the change in the modified Rodnan skin score (mRSS) and the change from baseline in forced vital capacity (FVC) in patients with SSc-associated ILD are key secondary endpoints. Biomarker assessments in DAISY include pharmacokinetics/pharmacodynamics, measurement of the whole-blood 21-gene type I interferon signature, and collection of serum/plasma and optional skin biopsies to explore the mechanism of action and its relationship to the clinical outcomes. Consistent with the prior phase I study of IFNAR blockade in SSc, which demonstrated rapid suppression of the type I interferon signature in blood and in skin after dosing, these analyses are expected to further elucidate anifrolumab’s mechanistic effects in lesional skin [10,54] (Table 1).

Additional translational evidence supporting interferon-pathway targeting in fibrosing autoimmune disorders comes from systemic lupus erythematosus and dermatomyositis, where IFN-I-driven transcriptional programs play a central role. In SLE, clinical trials demonstrated that anifrolumab reduced the overall disease activity and improved skin disease measures within SLE cohorts while maintaining an acceptable safety profile [47,49]. In dermatomyositis, therapeutic modulation of the JAK–STAT pathway has shown clinical benefit in proof-of-concept studies, accompanied by attenuation of type I interferon-induced molecular signatures; systematic evidence synthesis further supports the potential role of JAK inhibitors across reported dermatomyositis cohorts [55]. Given that SSc shares interferon-driven inflammatory networks with these conditions, particularly involving pDCs, MxA, and CXCL10, these parallel findings further strengthen the therapeutic rationale for IFN blockade in SSc.

Another layer of evidence arises from biomarker studies, which consistently identify high interferon signatures as predictors of aggressive disease and poor prognosis. SSc patients with elevated ISG expression have a faster progression of skin fibrosis, a higher prevalence of ILD, and increased cardiovascular complications [6]. These correlations suggest that the interferon signature could serve not only as a pharmacodynamic biomarker but also as a stratification tool to select patients most likely to benefit from IFNAR inhibition.

The results from preclinical and translational studies indicate that IFN-I signaling represents a key pathogenic link between innate immune activation, vascular injury, and fibroblast activation. Animal studies demonstrate that IFNAR blockade attenuates fibrosis and vascular remodeling, while evidence from lupus and dermatomyositis supports the feasibility and safety of sustained IFNAR inhibition. The ongoing phase III DAISY trial (NCT05925803) will determine whether these molecular and mechanistic insights translate into meaningful clinical benefit for patients with diffuse cutaneous SSc. However, the current evidence remains limited by small sample sizes, a short study duration, and the absence of published phase II or III results, underscoring the need for confirmatory clinical data. Despite a strong biological rationale and consistent translational evidence, clinical efficacy data for anifrolumab in SSc remain limited. Therefore, the current evidence should be regarded as preliminary and hypothesis-generating, highlighting the need for adequately powered randomized controlled trials to establish its therapeutic role. If successful, interferon-signature-guided therapy could initiate a new precision-medicine paradigm in SSc.

## 6. Comparison with Other Biologics or Targeted Agents in Systemic Sclerosis

The emergence of anifrolumab in SSc coincides with a broader therapeutic shift toward targeted immunomodulation based on defined molecular pathways. Unlike traditional immunosuppressants that broadly suppress immune activation, emerging biologics and small molecules in SSc selectively modulate specific pathways within the inflammatory–fibrotic network. Within this landscape, anifrolumab occupies a unique position by targeting the IFN-I axis, a central upstream regulator of both vascular and fibrotic pathology [2,10].

Among the most extensively studied agents is tocilizumab, a monoclonal antibody directed against the interleukin (IL)-6 receptor. The rationale for IL-6 blockade arises from the observation that IL-6 promotes fibroblast activation and endothelial injury through STAT3 signaling and crosstalk with IFN-inducible pathways [56]. In the faSScinate and focuSSced phase II/III trials, tocilizumab demonstrated trends toward improvement in skin fibrosis (mRSS) and the preservation of lung function, particularly in early diffuse cutaneous SSc [57]. Although the primary endpoint was narrowly missed in focuSSced, significant benefits in the forced vital capacity underscore its antifibrotic potential. Mechanistically, IL-6 and IFN-I share downstream activation of JAK–STAT signaling, suggesting that a dual pharmacological or biological blockade could yield additional benefits. Thus, anifrolumab’s upstream inhibition of IFNAR1 may indirectly attenuate IL-6-driven fibroblast responses, highlighting an IFN–IL-6 axis that links innate immunity to fibrosis [2].

Rituximab, a CD20-targeting monoclonal antibody that depletes B cells, has shown benefit in both observational cohorts and controlled studies in SSc. Key clinical datasets indicate that rituximab improves skin scores and may stabilize ILD with an acceptable safety profile [58,59]. Given the potential contribution of B cells to immune-complex-mediated activation of interferon pathways, rituximab may indirectly modulate interferon activity; however, anifrolumab provides a direct receptor-level blockade of type I interferon signaling and well-characterized pharmacokinetic/pharmacodynamic properties [38]. Combination or sequential use of B-cell depletion and IFNAR inhibition remains strictly hypothesis-generating, and the risk of additive immunosuppression warrants careful evaluation in clinical studies.

In contrast, romilkimab, a bispecific monoclonal antibody that neutralizes both IL-4 and IL-13, directly targets Th2-driven fibrotic pathways. In a phase II trial, romilkimab significantly reduced the mRSS and showed favorable trends in the quality-of-life outcomes. These cytokines function downstream of interferon-activated macrophages, driving fibroblast proliferation and collagen production. Thus, while romilkimab acts at the late effector stage of fibrosis, anifrolumab intervenes earlier in the cascade, potentially preventing the initiation of IL-4/IL-13-mediated remodeling [38,60]. This comparison highlights that targeting interferon signaling addresses earlier immune drivers of fibrosis, whereas IL-4/IL-13 inhibition acts at a downstream effector level.

Lenabasum, a selective cannabinoid receptor type 2 agonist, attenuates inflammation and reduces collagen deposition through the activation of pro-resolving lipid mediators. Early clinical studies demonstrated modest improvements in skin fibrosis; however, the phase III RESOLVE-1 trial did not meet its primary endpoint, likely reflecting patient heterogeneity and advanced disease stages. Despite these limitations, lenabasum exerts immunoregulatory and antifibrotic effects that could theoretically complement IFN-I inhibition, particularly in early disease when inflammation predominates [61]. Combining pro-resolving and anti-interferon approaches may therefore represent a promising future therapeutic strategy in SSc.

Nintedanib, a tyrosine kinase inhibitor approved for SSc-associated ILD, targets receptors for vascular endothelial growth factor, fibroblast growth factor and platelet-derived growth factor. Through this inhibition, it reduces fibroblast proliferation and migration [62]. Although its mechanism is mainly non-immunologic, transcriptomic studies have shown partial normalization of interferon- and transforming growth factor beta-related gene signatures in treated patients [63]. These findings suggest that antifibrotic and immunomodulatory treatments may influence overlapping molecular pathways, supporting the concept of the IFNAR blockade as a potential integrative therapeutic target.

The efficacy profiles of current predominant biologics indicate distinct therapeutic domains in SSc: tocilizumab and rituximab primarily regulate immune activation; romilkimab and nintedanib act on fibrotic remodeling; lenabasum enhances endogenous inflammation control; and anifrolumab uniquely blocks innate immune amplification through IFN-I inhibition. Unlike cytokine- or growth-factor-specific agents, IFNAR blockade may induce broader transcriptional changes, simultaneously suppressing multiple inflammatory and fibrotic mediators. This wide-ranging effect could be especially relevant in patients with a high interferon gene signature, which is increasingly recognized as a marker of diffuse and progressive disease, Santos et al. [2].

Beyond these established therapeutic strategies, the SSc treatment landscape continues to expand with the development of multiple targeted and investigational approaches aimed at distinct immune and fibrotic pathways [64,65,66]. Several ongoing clinical programs focus on modulating B-cell survival and activation, T-cell costimulatory signaling, intracellular inflammatory cascades, and vascular–fibrotic pathways, reflecting the multifaceted nature of SSc pathogenesis [64,67,68,69,70,71]. In parallel, exploratory immunomodulatory strategies seek to achieve deeper or more sustained immune control than conventional biologics, particularly in patients with aggressive or refractory disease [72].

Targeted inhibition of B-cell-driven pathways through BAFF or BAFF/APRIL blockade has gained increasing attention, with agents such as belimumab and telitacicept currently under clinical evaluation in SSc and SSc-associated ILD [64,71]. In addition, the modulation of immunoglobulin recycling via neonatal Fc receptor inhibition represents a novel strategy to reduce pathogenic IgG autoantibodies, although its clinical application in SSc remains at an early stage [72].

Selective intracellular signaling inhibitors are also being explored, including phosphodiesterase-4B inhibition and other small-molecule approaches evaluated within adaptive platform trial designs, enabling the parallel assessment of multiple therapies within a unified framework. In this context, nerandomilast and amlitelimab are being investigated within the CONQUEST platform trial, reflecting an increasingly efficient approach to evaluating targeted therapies across inflammatory and fibrotic disease domains [69].

Beyond pharmacologic agents, advanced immunotherapeutic strategies are under investigation, including CD19-directed cellular therapies and bispecific antibody platforms designed to simultaneously modulate immune activation and fibrotic effector pathways [2,72]. Although these approaches remain highly exploratory, early translational and clinical observations suggest the potential for profound immunomodulation in selected patients with severe or refractory disease, warranting further controlled investigation [72].

In summary, anifrolumab offers a distinct and potentially integrative mechanism within the growing landscape of SSc-targeted therapies. By modulating the upstream regulator of innate immune activation, it may complement or enhance the effects of IL-6, B-cell, or antifibrotic inhibitors. Future precision-medicine approaches combining molecular stratification with pathway-specific interventions, particularly within the IFN–IL-6–TGF-β axis, will clarify the optimal therapeutic role of anifrolumab in SSc. An overview of the currently available and investigational targeted agents in SSc is summarized in Table 2.

## 7. Future Directions and Challenges

The future clinical positioning of anifrolumab in SSc will depend on precise patient selection and integration with other targeted therapies. This development reflects the ongoing shift toward precision immunotherapy, where treatment efficacy relies on matching the therapeutic target to the patient’s dominant disease mechanism. Identification of the IFN-I gene signature provides a foundation for biomarker-based stratification, as high interferon activity is associated with diffuse skin disease, early inflammatory onset, and multiorgan involvement [73]. Utilizing this molecular signature to stratify patients could enrich clinical trial populations and ultimately improve the treatment effectiveness. However, the clinical implementation of interferon signature-guided therapy remains challenging due to the lack of standardized assays. Currently, IFN signatures are assessed using heterogeneous gene panels and analytical platforms, leading to variability across studies and clinical settings. This lack of harmonization represents a significant barrier to the routine adoption of biomarker-driven precision medicine approaches in SSc.

Unlike broad immunosuppressants, anifrolumab specifically modulates the innate immune activation that contributes to endothelial damage, macrophage stimulation, and fibroblast transformation. IFN-driven activity appears most pronounced in the early stages of the disease and may become less therapeutically relevant once irreversible fibrosis is established, suggesting that early intervention could be critical. This concept parallels findings in SLE, where anifrolumab demonstrated highest benefit in patients with high baseline interferon activity [45,49]. It is important to emphasize that, in this context, early refers to a disease phase dominated by active immune and vascular inflammation with limited irreversible fibrotic remodeling and elevated interferon activity, rather than disease duration alone, acknowledging the heterogeneous onset and progression of SSc.

In addition to the biological efficacy, the route of administration may influence the clinical utility of anifrolumab in SSc. Intravenous administration ensures predictable pharmacokinetics and complete bioavailability but requires regular hospital-based infusions, which may limit the long-term adherence. In contrast, subcutaneous formulations offer higher convenience and the potential for self-administration, which could improve the treatment adherence and patient satisfaction. While both routes achieve effective IFNAR blockade, real-world data will be needed to determine whether differences in the pharmacokinetics, dosing flexibility, and tolerability translate into clinically meaningful advantages in SSc [74].

Combination approaches may help address the complexity of immune–fibrotic crosstalk. Interactions between IFN-I, IL-6, and TGF-β sustain inflammation and fibrosis. Pairing anifrolumab with agents such as tocilizumab, nintedanib, or lenabasum could simultaneously target immune dysregulation and tissue remodeling [75]. When considering these potential combinations, it is essential to assess whether overlapping immunosuppression is clinically acceptable. Anifrolumab plus tocilizumab is conceptually compelling, because both target inflammatory pathways involved in early diffuse skin disease, with potential complementarity in patients who remain “highly inflamed” despite therapy [2,57]. The main limitation is safety: combining two immunomodulatory biologics would likely increase the risk of infection and other adverse events. Given the absence of data supporting their concomitant use, the risk–benefit ratio should therefore be carefully evaluated. Anifrolumab plus nintedanib may be more feasible from a safety perspective, because nintedanib is primarily an antifibrotic agent. This pairing is intuitively most appropriate for patients with SSc-associated ILD, where nintedanib slows the decline in FVC, while the IFNAR blockade could, in principle, address upstream immune activation that may contribute to permanent tissue damage. Potential limitations relate mainly to tolerability and adherence, including gastrointestinal adverse events and the need for liver enzyme monitoring, as well as uncertainty regarding the additive benefit in the absence of prospective data [63]. Anifrolumab plus lenabasum is theoretically attractive, because lenabasum is designed to promote the resolution of inflammation, which could make combination the with an IFNAR blockade more acceptable if efficacy is established. However, the clinical development in dcSSc has been limited by inconsistent efficacy signals, and the rationale, therefore, currently remains mechanistic rather than evidence based [61]. Overall, these combinations differ in plausibility and risk profile: the tocilizumab pairing raises the highest concern for additive immunosuppression, the nintedanib pairing is conceptually aligned with ILD and carries fewer immunological safety concerns, and the lenabasum pairing is biologically attractive but limited by clinical efficacy data. In all cases, optimal sequencing and safety profiles require evaluation in controlled studies before implementation in routine clinical care.

Organ-specific evaluation represents another frontier. While most SSc trials rely on skin endpoints, interferon activity also contributes to pulmonary vascular remodeling, myocardial inflammation, and microangiopathy [3,4,76]. Advanced imaging biomarkers, such as interferon-related molecular probes or AI-enhanced cardiac MRI, could enable the real-time assessment of IFN pathway activity and help identify early responders.

One of the key practical issues for the IFNAR blockade is the size and timing of the candidate population. Available transcriptomic and biomarker studies suggest that an elevated type I interferon gene signature is detectable in a substantial subset of SSc patients, particularly within inflammatory diffuse cutaneous phenotypes and in association with internal organ involvement in some cohorts [14,15,16,17,18,19]. However, the extent to which the proportion of “IFN-high” patients declines with increasing disease duration and whether interferon activity reliably transitions to a fibrosis-dominant state across individuals remains insufficiently defined by longitudinal human data, Brkic et al. [18]. Similarly, the likely duration of therapy required for a sustained clinical benefit has not yet been established. In SLE, pharmacodynamic studies demonstrate the rapid and durable suppression of interferon-stimulated gene expression after IFNAR blockade, but equivalent kinetics in SSc, particularly in skin- and organ-specific compartments, remain to be demonstrated [38,44,45]. These uncertainties highlight several priorities for future research: (1) defining clinically actionable interferon thresholds and harmonized assays for patient selection; (2) mapping longitudinal interferon-signature pathways across disease stages and treatment exposures; (3) determining whether early suppression of interferon activity translates into prevention of downstream vascular injury and fibrosis; (4) establishing treatment duration, monitoring intervals, and stopping criteria (including whether interferon signatures rebound after discontinuation and how this relates to relapse risk), Khanna et al. [10]. Addressing these issues is essential for translating interferon-guided therapy into a practical precision-medicine strategy in SSc.

From a safety perspective, anifrolumab’s tolerability in SLE is encouraging, but long-term data in fibrosing autoimmune diseases are limited. IFNAR blockade may increase the susceptibility to viral infections, most notably herpes zoster [48]. Experience from lupus can guide safety management in SSc. Importantly, IFNAR inhibition preserves type II and III interferon responses, maintaining protection against most pathogens [38]. Although serious opportunistic infections were uncommon, these findings underscore the importance of appropriate vaccination strategies, vigilant infection monitoring, and careful patient selection [49]. In SSc, where patients may already have significant organ involvement, dedicated safety data from ongoing trials are essential [10]. Continued safety monitoring tailored to SSc comorbidities, including pulmonary hypertension, renal crisis, and cardiac fibrosis, remains essential.

The potential impact of the IFNAR blockade on vascular homeostasis in these settings remains insufficiently characterized and warrants careful clinical monitoring. Furthermore, anifrolumab is likely to be used in combination with background immunosuppressive agents or antifibrotic therapies, raising important considerations regarding cumulative immunosuppression, infection risk, and drug–drug interactions. These factors highlight the need for integrated safety assessments in SSc-specific clinical trials and real-world practice. Insights from viral immunology further illustrate the complexity of type I interferon regulation. Seneca Valley virus has evolved multiple immune evasion strategies that suppress host innate immune responses, including direct interference with IFN-I signaling pathways [77]. Notably, the viral 3C protease has been shown to cleave optineurin (OPTN), thereby impairing selective autophagy and type I interferon signaling, highlighting a direct mechanism of IFN pathway antagonism [78].

An additional safety advantage of anifrolumab is the preservation of the host antiviral defense, as it does not affect type II interferon (interferon-γ) activity, which appears to contribute to the pathophysiology of SSc. Data from fibroblasts derived from patients with early dcSSc show that interferon-γ suppresses the expression of certain collagen genes, underscoring a dual and context-dependent role in inflammation and fibrosis [79]. These observations raise the question of whether selective modulation of interferon-γ-driven pathways might be beneficial in specific disease stages or molecular subgroups and how such approaches might interact with type I interferon blockade.

The next step toward personalized therapy will rely on integrated molecular profiling to refine disease classification. Combining transcriptomic, proteomic, and metabolomic data may distinguish inflammatory phenotypes such as “IFN-high”, “IL-6-high”, and “TGF-β-dominant” subsets [80]. This molecular stratification could improve both treatment selection and trial design, enabling smaller biomarker-driven studies. Adaptive platform trials, similar to those in oncology, may further accelerate the evaluation of targeted therapies such as anifrolumab.

Finally, defining treatment success in early SSc remains challenging. Reliance on the mRSS alone may underestimate meaningful biological improvement. Complementary endpoints, such as biomarker modulation, vascular stabilization, and prevention of organ involvement, could better capture the therapeutic benefit. Longitudinal assessment of the IFN gene signature, circulating CXCL10, and microvascular imaging may serve as sensitive indicators of disease control and the response to IFNAR inhibition.

## 8. Conclusions

SSc remains a therapeutic challenge driven by the interplay of immune activation, vascular injury, and fibrosis. IFN-I has emerged as a central mediator, linking innate immune dysregulation to fibroblast activation and microangiopathy. By selectively blocking the IFNAR1 subunit, anifrolumab offers a targeted strategy to interrupt this upstream pathogenic pathway. While interferon pathway inhibition represents a promising therapeutic strategy in systemic sclerosis, the available clinical evidence for anifrolumab remains preliminary. Definitive conclusions regarding its efficacy, optimal patient selection, and long-term safety await the results of ongoing randomized trials, particularly the phase III DAISY study.

## Figures and Tables

**Table 1:** Clinical studies of type I interferon blockade in systemic sclerosis.

Trial/Study	Population (*n*)	Study Design	Regimen	Estimated Effect/Key Findings	Safety Observations
MEDI-546 Phase I [54]	Systemic sclerosis (*n* = 34)	Phase I, multicenter, open-label, dose-escalation	Intravenous MEDI-546 (single and multiple ascending doses)	Dose-dependent suppression of interferon-regulated gene expression; confirmed pharmacodynamic target engagement	Generally well tolerated; upper respiratory tract infections were most frequently reported
DAISY (NCT05925803) [10]	Diffuse cutaneous SSc (planned *n* ≈ 300)	Phase III, randomized, double-blind, placebo-controlled	Subcutaneous anifrolumab vs. placebo	Primary endpoint: rCRISS25 at Week 52; secondary endpoints include mRSS, FVC, and organ-specific outcomes	Ongoing; safety monitoring with focus on infection risk

Abbreviations: FVC, forced vital capacity; mRSS, modified Rodnan skin score; rCRISS25, Revised Composite Response Index in Systemic Sclerosis 25; SSc, systemic sclerosis.

**Table 2:** Current targeted and antifibrotic therapies in systemic sclerosis.

Drug	Target/Pathway	Mechanism of Action	Ref.	Main Clinical Outcomes	Status	Key Safety Considerations
Anifrolumab	Type I interferon receptor (IFNAR1)	Monoclonal antibody blocking type I interferon signaling	[10]	CRISS-based composite outcome; lung function, skin and PROs	Phase III, ongoing	Viral infections, particularly herpes zoster, reported with IFNAR blockade; SSc-specific safety outcomes pending.
Tocilizumab	IL-6 receptor	Monoclonal antibody inhibiting IL-6–mediated inflammation and fibrosis	[57]	Preservation of FVC in SSc-ILD; inconsistent skin benefit	Approved for SSc-ILD (selected regions)	Increased risk of infections; laboratory abnormalities including neutropenia and elevated liver enzymes.
Rituximab	CD20	Anti-CD20 monoclonal antibody inducing B-cell depletion	[59]	Improved mRSS and lung function stabilization	Off label; supported by RCTs	Infusion reactions and infections; risk of hypogammaglobulinemia with repeated treatment.
Belimumab	BAFF (BLyS)	Monoclonal antibody inhibiting soluble BAFF and B-cell survival	[64]	Pulmonary function and safety outcomes in SSc-ILD with mRSS evaluation	Phase II/III, ongoing	Safety outcomes not yet reported for SSc-ILD trials (ongoing).
Romilkimab (SAR156597)	IL-4/IL-13	Bispecific monoclonal antibody neutralizing IL-4 and IL-13	[60]	Significant improvement in mRSS vs. placebo	Phase II completed	Generally acceptable safety profile; infections and injection-related reactions reported.
Lenabasum	CB2 receptor	Oral cannabinoid receptor type-2 agonist with pro-resolving effects	[61]	Failed to meet primary CRISS endpoint	Development discontinued	Generally well tolerated with predominantly mild to moderate adverse events.
Nintedanib	PDGFR/FGFR/VEGFR	Tyrosine kinase inhibitor with antifibrotic activity	[63]	Reduced annual rate of FVC decline in SSc-ILD	Approved for SSc-ILD	Gastrointestinal adverse events, particularly diarrhea; liver enzyme elevations requiring monitoring.
Guselkumab	IL-23 (p19)	Monoclonal antibody inhibiting IL-23 signaling	[67]	Change from baseline in mRSS	Phase II completed	Safety outcomes not yet reported for SSc trials referenced.
Ianalumab (VAY736)	BAFF-R	Anti-BAFF receptor monoclonal antibody causing B-cell depletion	[68]	Achieving 3/5 rCRISS25	Phase II, ongoing	Safety outcomes not yet reported; trial ongoing.
Amlitelimab (KY1005)	OX40–OX40L axis	Non-depleting anti-OX40L monoclonal antibody reducing T-cell activation	[69]	Changes in FVC and overall treatment response (rCRISS)	Phase IIb, ongoing	Safety outcomes not yet reported in the SSc platform trial context.
Nerandomilast (BI 1015550)	PDE4B	Selective phosphodiesterase-4B inhibitor with anti-fibrotic effects	[69]	Changes in FVC and overall treatment response (rCRISS)	Phase IIb, ongoing	Safety outcomes not yet reported in the SSc platform trial context.
Avenciguat (BI 685509)	sGC	Oral soluble guanylate cyclase modulator enhancing NO–cGMP signaling	[70]	Changes in FVC, mRSS, CRISS, HAQ-DI and vascular outcomes	Phase II, ongoing	Safety outcomes not yet reported; phase II trial ongoing.
Telitacicept	BLyS/BAFF and APRIL	Recombinant fusion protein comprising TACI receptor and human IgG Fc	[71]	Changes in mRSS and lung function; efficacy and safety in dcSSc	Phase II, recruiting	Safety outcomes not yet reported; clinical trial currently recruiting.
CAR-T19	CD19 (CAR-T)	Autologous CAR-T cell therapy inducing B-cell depletion	[72]	Effects on fibrotic and vascular organ manifestations in dcSSc	Early exploratory clinical experience	Cytokine release syndrome and cytopenias; increased infection risk during immune reconstitution.
MT-7117	Melanocortin receptor modulation	Anti-inflammatory and anti-fibrotic effects	[65]	Efficacy using ACR CRISS	Phase II completed	Safety outcomes not yet reported for the referenced phase II study.
Tulisokibart (MK-7240/PRA023)	TL1A	Humanized monoclonal antibody directed against TL1A	[66]	Efficacy and safety in SSc-ILD	Phase II, active	Safety outcomes not yet reported; phase II study ongoing.
Bispecific antibodies (bsAbs)	Dual immune/fibrotic targets	Simultaneous targeting of two immune or fibrotic pathways	[2]	No mature SSc-specific efficacy data	Preclinical	No mature SSc-specific clinical safety data; safety expected to be target-dependent.

Abbreviations: ACR CRISS, American College of Rheumatology Composite Response Index in Diffuse Systemic Sclerosis; APRIL, a proliferation-inducing ligand; BLyS/BAFF, B-Lymphocyte Stimulator/B-Cell Activating Factor; CAR-T, chimeric antigen receptor T-cell therapy; CRISS, Combined Response Index for Systemic Sclerosis; dcSSc, diffuse cutaneous systemic sclerosis; FVC, forced vital capacity; HAQ-DI, Health Assessment Questionnaire: Disability Index; IgG, immunoglobulin G; IFNAR1, type I interferon receptor subunit 1; IL, interleukin; SSc-ILD, systemic sclerosis: interstitial lung disease; mRSS, modified Rodnan skin score; NO–cGMP, Nitric Oxide–Cyclic Guanosine Monophosphate; PDE4B, phosphodiesterase-4B; PDGFR/FGFR/VEGFR, Platelet-Derived Growth Factor Receptor/Fibroblast Growth Factor Receptor/Vascular Endothelial Growth Factor Receptor; rCRISS, Revised Composite Response Index in Systemic Sclerosis 25 (rCRISS25); sGC, soluble guanylate cyclase; TACI, transmembrane activator and calcium modulator and cyclophilin ligand interactor; TL1A, tumor necrosis factor (TNF).

## Data Availability

No new data were created or analyzed in this study. Data sharing is not applicable to this article.

## Abbreviations

The following abbreviations are used in this manuscript:

CRISS	Composite Response Index in Systemic Sclerosis
EBV	Epstein–Barr virus
FVC	forced vital capacity
IFN	interferon
IFN-I	type I interferons
IFNAR	interferon α/β receptor
IFNAR1	interferon α/β receptor subunit 1
IL	interleukin
ILD	interstitial lung disease
IRFs	interferon regulatory factors
ISG	interferon-stimulated gene
ISGF3	interferon-stimulated gene factor 3
JAK/STAT	Janus kinase–signal transducer and activator of transcription
mRSS	modified Rodnan skin score
mtDNA	mitochondrial DNA
MxA	myxovirus-resistance protein A
pDCs	plasmacytoid dendritic cells
SLE	systemic lupus erythematosus
SSc	systemic sclerosis
TGF-β	transforming growth factor beta
TLRs	Toll-like receptors
